# Synergistic Effects of Endolift With Complementary Cosmetic Procedures: A Narrative Review and Comprehensive Approach

**DOI:** 10.1111/jocd.70922

**Published:** 2026-05-19

**Authors:** Mohammad Ali Nilforoushzadeh, Pantea Bozorg Savoji, Tannaz Fakhim, Shohreh Rafiei, Seyedeh Nasim Mirbahari

**Affiliations:** ^1^ Skin Repair Research Center Jordan Dermatology and Hair Transplantation Clinic Tehran Iran; ^2^ Skin and Stem Cell Research Center Tehran University of Medical Sciences Tehran Iran

**Keywords:** collagen remodeling, Endolift, laser‐assisted contouring, non‐surgical aesthetic procedures, rejuvenation

## Abstract

**Background:**

The landscape of aesthetic medicine has increasingly favored minimally invasive interventions, with the 1470 nm diode laser (Endolift) gaining prominence as a viable alternative to surgical lifting for soft‐tissue remodeling and lipolysis.

**Aims:**

This narrative review synthesizes current evidence on the mechanism, clinical versatility, and safety profile of Endolift. A primary objective is to evaluate the synergistic potential of combining this technology with complementary aesthetic modalities to optimize patient outcomes.

**Patients/Methods:**

A comprehensive search of PubMed/MEDLINE, Scopus, and Google Scholar was conducted. We analyzed key literature on the 1470 nm diode laser, selecting studies that document its efficacy as monotherapy and in combination with injectables or regenerative techniques.

**Results:**

The 1470 nm wavelength exhibits high affinity for water, facilitating selective lipolysis and stimulating deep collagen restructuring through controlled photothermal interaction. Clinical findings consistently report tangible improvements in skin laxity and contouring with limited recovery time. Notably, multimodal protocols—integrating dermal fillers, nanofat, or surface resurfacing—appear to enhance aesthetic outcomes by simultaneously addressing volume deficits and superficial texture alongside tissue tightening.

**Conclusions:**

Endolift represents a robust, minimally invasive tool for facial and body sculpting. While current data support its efficacy, particularly within multimodal treatment plans, the standardization of protocols through larger, randomized controlled trials remains a priority to confirm long‐term durability.

## 1 Introduction

**FIGURE 1:**
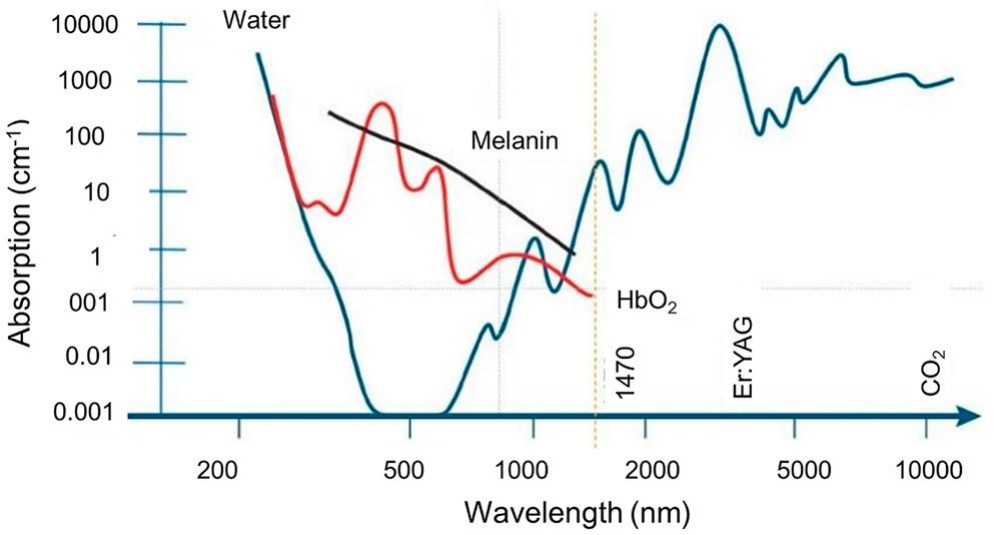
The graph illustrates the absorption of light by key tissue components, crucial for understanding the Endolift procedure. Endolift utilizes a laser, often with a wavelength of 1470 nm, which, as shown, is highly absorbed by water. This targeted absorption is essential in Endolift because the laser's energy is primarily delivered to the water within the subdermal tissue, causing a controlled thermal effect. This thermal stimulation leads to collagen contraction and tightening of the skin, the desired outcome of the Endolift procedure. The laser apparatus is fitted with a fractional scanner that is compatible with both 200 and 400‐μm power cables. By utilizing a cannula, which is introduced through a tab incision into the dermal layer, reaching the interface between the deep dermis and the superficial layer of subcutaneous adipose tissue, the surgeon is enabled to acquire multiple vectors of traction [2]. The graph also shows that melanin and hemoglobin absorb light in different ranges, which is important to consider to minimize unwanted side effects during the treatment. Ideally, the 1470 nm wavelength targets water while minimizing absorption by these other chromophores. Understanding these absorption characteristics allows practitioners to optimize the Endolift procedure for effective tightening and rejuvenation with minimal downtime and risk.

In recent years, aesthetic medicine has undergone a major shift toward minimally invasive procedures that deliver effective and long‐lasting results without the risks and downtime linked to traditional surgery. Among these advancements, Endolift has become a leading laser‐based technology that has transformed facial and body contouring [1]. Endolift provides a non‐surgical option for skin tightening, collagen remodeling, and localized fat reduction, making it appealing for those seeking skin rejuvenation with minimal recovery time. The 1500 LASEMAR diode laser system operates at 1470 nm, a wavelength optimized for high water absorption while reducing interaction with melanin and hemoglobin, ensuring precise tissue targeting (Figure 1). The laser source is a gallium arsenide (GaAs) semiconductor with adjustable settings for power (W), pulse duration (Ton/Toff), and fiber type (300, 400, 600, 1000 μm, etc.). The device offers continuous and pulsed emission modes, with fan‐shaped retrograde movement for even energy distribution. When the laser energy converts to thermal energy, it triggers a series of physiological reactions, including disruption of adipocyte integrity, immediate contraction of connective septa leading to tissue tightening, restoration of elastic fiber composition, neocollagenesis that promotes the formation of Type I and Type III collagen over time, and significant rejuvenation of the dermis where aging collagen fibers are gradually replaced by new ones [3] Its main uses include skin tightening, lipolysis, tissue coagulation, and resurfacing [4]. Originally developed for facial rejuvenation, Endolift has now expanded to treat various body areas, including skin laxity, stubborn fat deposits, aging concerns, and facial remodeling [5]. Studies, including those by our team, have shown the valuable clinical benefits of Endolift in reducing wrinkles, sagging skin, and resistant fat, especially in delicate regions like under the eyes, double chin, and neck. By stimulating tissue regeneration and improving dermal architecture, this laser‐assisted procedure enhances skin firmness and encourages natural collagen production, leading to long‐term improvements in skin texture and elasticity. While Endolift is highly effective alone, combining it with other cosmetic procedures can boost its effects, resulting in better aesthetic results. Multiple combination therapies are becoming more popular in aesthetic practice. The reasoning behind combining these procedures is that they target different skin layers and tissues, optimize collagen synthesis, and extend the effects of skin rejuvenation. While Endolift stimulates deep dermal remodeling, other techniques such as injectables, resurfacing lasers, and regenerative therapies improve volume, hydration, and skin tone. This article discusses how Endolift works, its benefits, and clinical outcomes, including its role in combination treatments, how it influences collagen production and fat reduction, and the specific facial and body areas where it has shown notable results backed by studies. It also explores the synergistic effects of combining Endolift with other cosmetic treatments to improve skin tightening, wrinkle reduction, and body contouring. Furthermore, it includes criteria for patient selection, risks, contraindications, and post‐treatment expectations. By understanding the science behind Endolift and its clinical applications, this article aims to provide useful insights for dermatologists, plastic surgeons, and aesthetic practitioners, as well as individuals interested in non‐invasive skin rejuvenation and body sculpting options. As aesthetic medicine advances, Endolift‐based combination therapies stand out as a promising step forward in achieving youthful, firm, and well‐contoured skin—without invasive surgery.

## 2 Methods

To ensure a comprehensive analysis of the interstitial laser lifting technique (Endolift), a narrative review of the literature was conducted. We performed an electronic search across primary medical databases, including PubMed/MEDLINE, Scopus, Web of Science, and Google Scholar. The search strategy used Boolean operators with the following keywords: “Endolift,” “1470 nm diode laser,” “interstitial laser lifting,” “subdermal laser,” “laser lipolysis,” and “minimally invasive facial contouring.” We prioritized clinical trials, prospective and retrospective cohort studies, case series, and relevant review articles published in the English language. The inclusion criteria focused on studies detailing the mechanism of action, clinical efficacy, safety profile, and specific protocols involving the 1470 nm diode laser. Special attention was given to recent literature (2020–2024) discussing synergistic approaches and combination protocols with other aesthetic modalities. Studies lacking specific laser parameters or utilizing wavelengths other than 1470 nm for similar indications were excluded to maintain focus on the specific technology under review.

## 3 Mechanism of Action and Tissue Interaction

The fundamental mechanism of the Endolift procedure relies on the selective delivery of laser energy through a micro‐optical fiber inserted into the subcutaneous tissue. The system utilizes a 1470 nm diode laser, a wavelength specifically selected for its high absorption coefficient in water and fat, which are the primary chromophores in the target tissue [6, 7].

According to recent studies, the interaction between the laser beam and the tissue produces a controlled photothermal effect [6, 8]. When the optical fiber delivers energy to the superficial hypodermis, two distinct physical phenomena occur simultaneously:

1. Selective Lipolysis: The thermal energy targets adipocytes, inducing the emulsification of localized fat deposits. This is particularly relevant for contouring areas with stubborn adipose tissue, as the laser energy disrupts the fat cell membranes [6, 7].
2. Collagen Remodeling and Septal Retraction: The heat generated by the interaction with tissue water causes immediate contraction of the collagen fibers within the fibrous septae. This shrinkage effect provides immediate tissue retraction [7, 8]. Furthermore, the thermal stress triggers a wound‐healing response in the dermis, stimulating fibroblasts to synthesize new collagen (neocollagenesis) and extracellular matrix components over the weeks following the procedure [6, 7].

Research by Sadoughifar et al. [8] and Markabayeva et al. [7] on cutaneous ptosis highlights that this Direct Optical Energy allows for tightening of lax skin without the need for surgical excision. The energy is delivered interstitially, bypassing the epidermis, which minimizes surface thermal damage while maximizing deep tissue heating. Additionally, Medhat et al. [9] demonstrated the versatility of this interstitial laser mechanism in treating deeper structural conditions, such as hidradenitis suppurativa, by effectively targeting and destroying specific follicular and tract structures through precise thermal coagulation.

**FIGURE 2:**
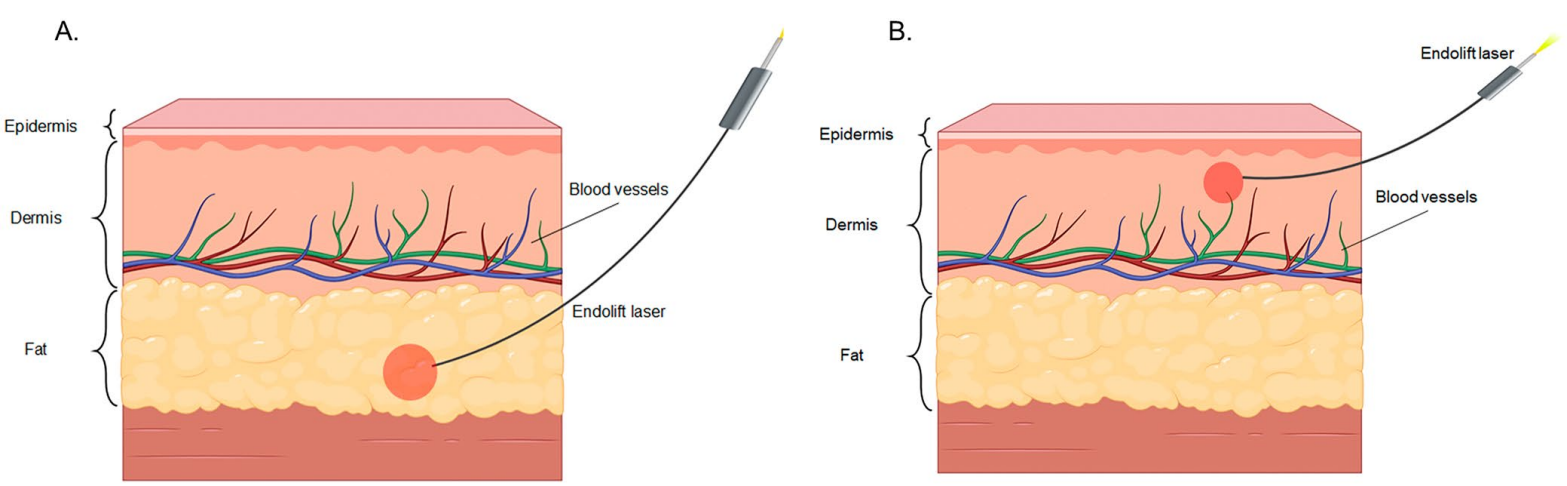
Schematic representation of the two applications of the Endolift laser. (A) Deep tissue targeting for skin tightening and fat reduction. (B) Superficial tissue targeting to stimulate collagen production and enhance skin rejuvenation.

Collectively, these mechanisms, such as lipolysis, immediate septal retraction, and delayed neocollagenesis, result in the clinical observation of skin tightening and volume reduction described in the literature [6, 7, 8, 9]. A schematic comparison of these two tissue interaction levels is illustrated in Figure 2.

## 4 Procedural Technique and Guidelines

**FIGURE 3:**
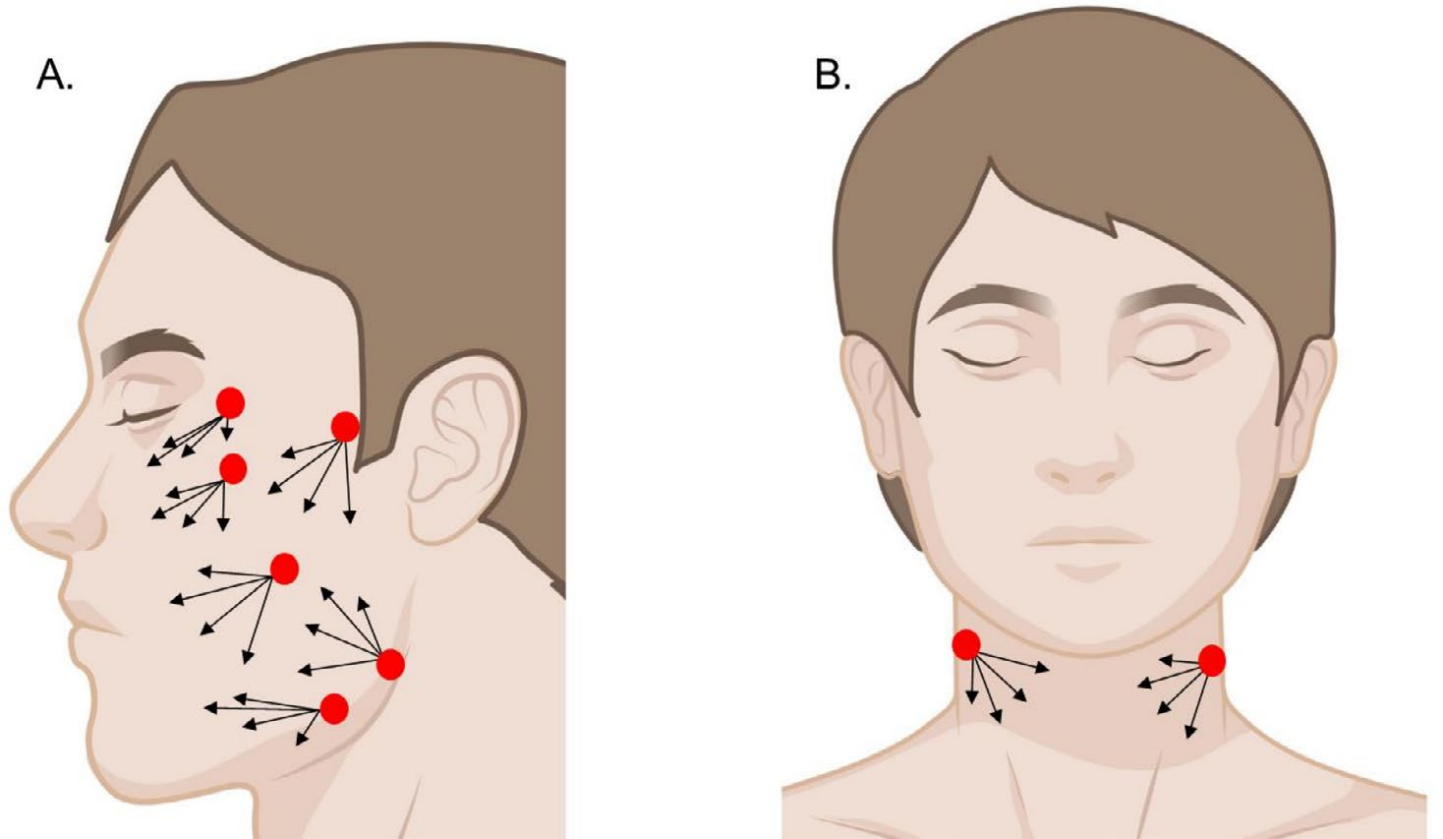
Endolift procedures target specific anatomical points to achieve facial and neck rejuvenation. In facial applications (A), entry sites are strategically located to address concerns such as midface volume loss, nasolabial folds, and jowl laxity. Vectors depicted indicate the direction of laser fiber insertion for targeted tightening and lifting effects. Similarly, for neck contouring (B), entry points are positioned to improve definition along the jawline and neck. These precise entry and vector strategies are crucial for delivering energy effectively and achieving optimal aesthetic outcomes in Endolift procedures.

Endolift technology is applied through specific entry points depending on the targeted facial area. For the upper face, including the forehead and crow's feet, access is typically through the temporal region near the hairline to treat the delicate skin around the eyes and brow. In the mid‐face, particularly the cheeks, entry is at the nasolabial folds or just below the cheekbone, allowing for deeper collagen stimulation. For the lower face, including the jawline and neck, the preferred access points are beneath the chin or along the jawline to enhance contour and address sagging skin. Each entry is strategically chosen to ensure precise energy delivery to targeted skin layers while minimizing discomfort and downtime. The clinical endpoint is determined by changes in tissue consistency, with the area becoming softer and more uniform in texture, along with visible and tactile alterations in shape. These findings are based on 20 years of patient management experience reported by Dr. Nilforoushzadeh et al. A well‐distributed erythema and noticeable firming of the treated area indicate an optimal response, whereas excessive thickening and a shift toward white discoloration suggest overtreatment, which may lead to skin peeling and should be avoided. While the total delivered energy is an important factor, it is a secondary reference since treatment parameters should be tailored to individual patient characteristics. Maintaining symmetry across both sides of the face is vital, and using lower power over a longer duration can produce results that are comparable or even superior to high‐power settings, which may limit energy diffusion and reduce tissue tightening. As a guideline, the standard energy requirement for a 10 × 10 cm treatment area is approximately 1000 joules. Figure 3 illustrates the common entry points and treatment vectors for this minimally invasive procedure.

The fiber is inserted into the superficial hypodermis using a retrograde fan‐shaped movement to achieve uniform energy dispersion. Settings vary based on the treated area and indication, including skin tightening (300 μm fiber, 2.5–3.5 W, 25%–30% Ton), lipolysis (600 μm fiber, 4.0–6.0 W, 40%–50% Ton), and collagen remodeling, which requires customized settings based on the patient's specific needs. Each treatment area—such as the eyes, face, neck, body, legs, and arms—requires tailored power and pulse settings to ensure optimal results. The device's real‐time energy monitoring enhances safety and effectiveness, minimizing risks while maximizing treatment precision. Table 1 outlines the recommended settings for a treatment procedure based on different treatment areas, skin types, and specific parameters such as pulse duration (Ton), pulse off‐time (Toff), and power (W). It provides tailored settings for areas like the eyes, face, and neck, considering variations in skin thickness and the presence of fat. For example, the settings for the eyes are lower in power, while the face and neck areas have different recommendations depending on whether the skin is thin or thick, and whether fat is present, with power settings ranging from 1.5 to 4 W to achieve optimal treatment outcomes. Proper post‐treatment care is crucial for enhancing results and ensuring patient comfort, with recommended measures including cold compression to reduce swelling, avoiding excessive heat exposure (such as saunas and direct sun), and gentle skincare routines to support skin healing and regeneration. With these precise protocols, Endolift provides consistent and safe outcomes, reinforcing its role as a leading innovation in aesthetic medicine.

**TABLE 1:** Recommended settings for a treatment procedure based on different treatment areas, skin types, and specific parameters such as pulse duration (Ton), pulse off‐time (Toff), and power (W).

Treatment area	Fiber diameter (μm)	Power (W)	^a^ Ton (%)^b^	Toff (%)	Indication	Special notes
Periorbital/Eyes	300	1.5–2.0	20–25	75–80	Skin tightening, collagen remodeling	Use low power to avoid thermal damage; suitable for thin skin; avoid orbital fat pad penetration
Face (thin skin, minimal fat)	300–400	2.0–3.0	25–30	70–75	Collagen remodeling, skin tightening	Maintain superficial hypodermis plane; avoid over‐treatment to prevent epidermal injury
Face (thick skin or with fat)	400–600	3.0–4.0	25–30	70–75	Combined tightening and mild lipolysis	Multiple passes may be required for uniform tightening
Neck (Skin tightening)	300–400	2.5–3.5	25–30	70–75	Laxity reduction	Maintain symmetry across sides; retrograde fan‐shaped movement
Neck (with submental fat)	600	4.0–5.0	40–50	50–60	Lipolysis + tightening	Focus on even energy distribution; avoid the marginal mandibular nerve zone
Lower face/Jawline contouring	400–600	3.5–4.5	30–40	60–70	Jowl fat reduction, mandibular definition	Entry from submental or preauricular points; maintain uniform tissue heating
Abdomen (localized fat + laxity)	600–1000	4.0–6.0	40–50	50–60	Fat reduction + skin tightening	Larger treatment grid; approx. 1000 J per 10 × 10 cm area
Arms/Thighs	600–1000	4.0–5.0	40–50	50–60	Fat reduction, skin firming	Use multiple passes; adapt depth based on subcutaneous thickness
General collagen remodeling (any area)	Variable (300–600)	Custom^b^	25–40	60–75	Dermal rejuvenation	Parameters adjusted per patient's skin type, age, and clinical response

^a^ Ton (%) refers to the percentage of the pulse‐on time within the duty cycle.

^b^ Customized settings should consider skin phototype, dermal thickness, and previous aesthetic treatments.

## 5 Safety and Danger Zones and Clinical Considerations

Despite its benefits, Endolift is contraindicated in patients with metallic implants, active infections, pregnancy, coagulation disorders, skin diseases, and malignancies. Potential side effects may include bruising, swelling, transient neuropraxia, and mild discomfort, which typically resolve within a few days. To ensure patient safety and optimal results, pre‐procedural sterilization is crucial. This includes using disposable optical fibers, antiseptic solutions, protective eyewear, and adherence to strict sterile techniques. Depending on the treatment area and patient sensitivity, anesthesia options range from topical lidocaine to local infiltration or nerve blocks. During the procedure, the laser should be applied at the superficial hypodermis, ensuring proper temperature monitoring and tissue reaction assessment throughout. The fan‐shaped retrograde movement technique is used for even energy distribution, preventing excessive heating or damage to surrounding tissues.

**FIGURE 4:**
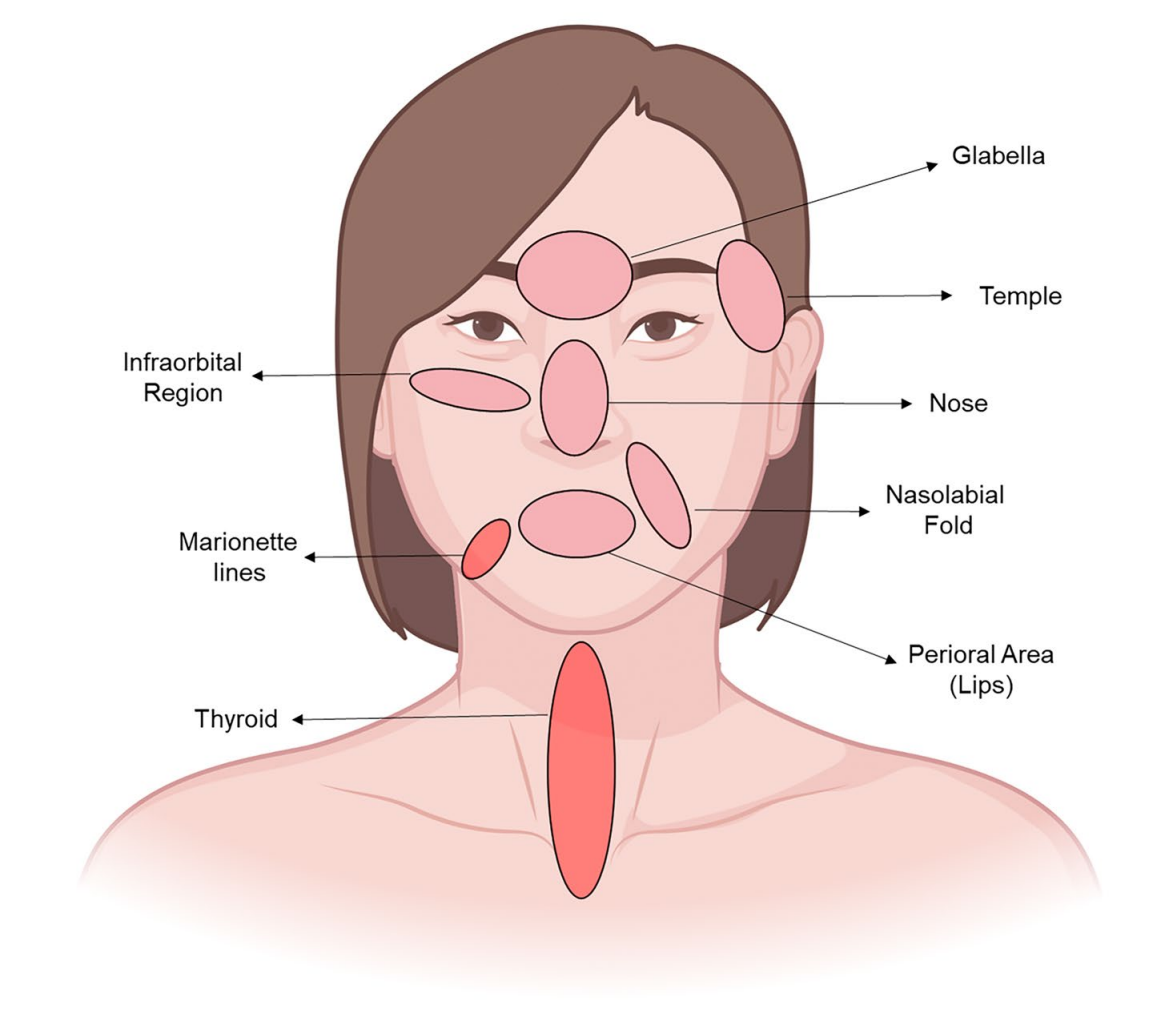
Schematic representation of Endolift laser application and caution zones. Pink circles indicate regions requiring caution during Endolift treatment, including the glabellar region, temples, infraorbital area, nose, perioral area (lips), and nasolabial folds. The red areas, representing the thyroid and marionette lines, are designated as forbidden zones due to potential risks. Precise technique and in‐depth knowledge of facial anatomy are crucial for minimizing the risk of complications, such as nerve damage, hematoma, burns, or contour irregularities, in these highlighted areas.

The Endolift procedure requires special attention to certain areas of the face and neck, referred to as the “danger zones,” due to the presence of delicate anatomical structures such as nerves, blood vessels, and muscles that are vulnerable to injury. These areas include the glabellar region, temples, infraorbital area, nose, perioral area (lips), and nasolabial folds [10]. Care must be taken in these regions to avoid potential complications. Additionally, the marionette lines and areas in front of the thyroid on the neck are prohibited for fiber insertion. It is essential to avoid these zones and exercise extra caution in regions near nerves. Temporary damage to the upper end of the marginal nerve can lead to temporary numbness (neuropraxia), which usually resolves within 2–4 weeks. Hypesthesia and paresthesia may also occur temporarily for a shorter duration. In these sensitive regions, it is recommended to release energy only in a retrograde manner to minimize the risk of nerve damage and ensure a safe and effective treatment (Figure 4). To minimize risks in these sensitive areas, it is recommended to release energy only in a retrograde manner, use the correct depth and energy settings, and apply controlled movements. A thorough understanding of facial anatomy, proper patient assessment, and adherence to safe techniques are essential for achieving optimal results while minimizing complications. For this reason, Endolift should only be performed by trained professionals with expert knowledge of facial and body anatomy, ensuring both safe and effective treatments.

## 6 Clinical Applications and Outcomes

The versatility of the 1470 nm diode laser allows for treatment across various anatomical regions, with protocols adjusted for local skin thickness and fat distribution.

The periorbital region is particularly responsive to Endolift due to the thinness of the skin. Studies have demonstrated its efficacy in treating lower eye bags [11, 12], upper eyelid laxity, and eyebrow ptosis [13, 14], offering a “lunch‐time” alternative to blepharoplasty with reduced downtime. For the upper face, the laser has been effectively used to smooth forehead wrinkles and static glabellar lines [15, 16], often serving as an adjunct to neurotoxins for deep static creases.

In the mid and lower face, Endolift targets heavy jowls and the submental area. Clinical evaluations have shown significant biometric reductions in jowl fat volume [17] and improvement in nasolabial folds and marionette lines [18]. In the neck, the procedure addresses horizontal wrinkles (Venus rings) and laxity, with biometric measurements confirming skin retraction and improved cervicomental angles [19].

Beyond the face, applications have expanded to body contouring. Recent evidence highlights the laser's utility in treating localized adiposity and laxity in the arms and lower abdomen [20]. In these areas, the lipolytic effect is prioritized, often requiring higher power settings compared to facial protocols [20, 21].

## 7 Discussion

Endolift has demonstrated significant efficacy as a standalone modality for lipolysis and photothermal skin retraction, as detailed in the mechanism of action. However, the complexity of facial aging, which involves bone resorption, deep fat deflation, and superficial textural changes, often exceeds the capabilities of a single device. Consequently, the current trend in aesthetic medicine favors multimodal approaches. This section discusses the specific synergistic roles of Endolift in combination therapies, weighing their advantages and limitations.

### 7.1 Endolift and Dermal Fillers (HA and CaHA)

Recent protocols, such as those described by Ilaria et al. [3], advocate for the combined use of Endolift with Hyaluronic Acid (HA) or Calcium Hydroxyapatite (CaHA).

The primary rationale is the “lift and fill” concept. Endolift provides the “scaffold” tightening by retracting the septal network and reducing heavy fat compartments (e.g., jowls), which alters the facial vectors. This allows for a more conservative use of dermal fillers to restore volume in deflated areas (e.g., midface), preventing the overfilled syndrome. The thermal stimulation of Endolift combined with the biostimulatory properties of CaHA may offer a synergistic boost to neocollagenesis [3].

The timing of the application is debated. Simultaneous application requires precise placement to avoid thermal degradation of the filler material or excessive edema. There is also a theoretical risk that laser‐induced inflammation could alter the rheological properties of HA fillers if injected into the same plane immediately [3].

### 7.2 Endolift and Regenerative Therapies (Nanofat and PRP)

The integration of Endolift with autologous nanofat grafting represents a convergence of thermal and biological stimulation.

As highlighted in studies on glabellar lines and deep creases [22], this combination addresses both structural laxity and skin quality. Endolift induces a controlled thermal injury that triggers a wound‐healing cascade, while nanofat provides adipose‐derived stem cells (ADSCs) and growth factors. This biological input optimizes the healing environment, potentially reducing fibrosis and enhancing the quality of the newly formed collagen [22, 23].

The viability of the grafted fat cells is a primary concern. High thermal energy from the laser could potentially compromise adipocyte survival if the procedures are performed in immediate proximity without adequate spacing or layer differentiation. Additionally, the procedure becomes more invasive, requiring a donor site for fat harvesting, which increases the total procedure time and recovery [22].

### 7.3 Endolift and Surface Resurfacing (Fractional Lasers/Microneedling)

While Endolift targets the deep dermis and subcutaneous interface, it has limited effects on epidermal pathology such as pigmentation or fine superficial lines.

Combining Endolift with fractional CO2 lasers or Radiofrequency (RF) microneedling creates a “sandwich” effect, treating the full thickness of the skin [23, 24]. The Endolift tightens the foundation, while the resurfacing modality addresses the canvas (epidermis), improving texture and pore size. This is particularly effective for acne scarring, where deep tethering is treated with the fiber, and surface irregularities are smoothed with resurfacing [23].

This combination significantly increases post‐procedural inflammation, edema, and downtime compared to Endolift alone. Patient selection must be rigorous, as the cumulative thermal injury increases the risk of hyperpigmentation, particularly in higher Fitzpatrick skin types [10, 25].

### 7.4 General Limitations of Combination Approaches

Despite the synergistic benefits, multimodal treatments introduce variables that complicate standardized protocols. The heterogeneity in laser settings, filler types, and patient anatomical variations makes it difficult to establish universal guidelines [4, 10]. Furthermore, combining modalities increases the complexity of adverse event management; for instance, distinguishing between laser‐induced neuropathy and cannula‐induced trauma from filler injection can be challenging [25, 26]. Future research must focus on defining the optimal sequencing, whether simultaneous or staged, to maximize safety and efficacy [4].

## 8 Conclusion

This narrative review has synthesized current evidence regarding the safety, efficacy, and clinical applications of the 1470 nm diode laser (Endolift). The literature supports the conclusion that interstitial delivery of laser energy effectively bridges the gap between non‐invasive topical treatments and surgical interventions, providing measurable skin tightening and lipolysis through a dual photothermal mechanism [4, 11, 15, 16, 17, 19, 21].

Consistent with the objectives outlined in the introduction, this review highlights that while Endolift is effective as a standalone modality for mild to moderate laxity, its clinical utility is significantly expanded when integrated into multimodal treatment plans. The data suggest that combining Endolift with volumetric restoration (fillers, nanofat) and surface resurfacing creates synergistic outcomes that address the multifactorial nature of aging, structural, volumetric, and textural more effectively than single‐modality approaches [3, 22, 23].

However, this review also identifies limitations within the current body of evidence. The majority of included studies are case series or retrospective analyses with relatively small sample sizes and short follow‐up periods [4, 10]. Furthermore, the heterogeneity in reported laser parameters such as energy settings, fiber diameter, and the reliance on subjective patient satisfaction scores in many studies complicates the establishment of standardized global protocols [10].

Future research should prioritize large‐scale, prospective, randomized controlled trials (RCTs) utilizing objective biometric measurements (such as 3D volumetric analysis) to validate the long‐term durability of these results. Additionally, comparative studies are needed to define optimal sequencing for the combination therapies discussed in this review. Ultimately, Endolift represents a versatile tool in the aesthetic armamentarium, but its evolution from a novel technique to a gold‐standard protocol requires a continued shift toward higher‐level evidence and standardization [4, 24, 25].

## Author Contributions

**Mohammad Ali Nilforoushzadeh:** conceptualization, supervision, project administration, and funding acquisition. **Pantea Bozorg Savoji:** methodology, writing original draft, data visualization, and investigation. **Tannaz Fakhim:** validation, literature review, and review and editing. **Shohreh Rafiei:** validation, literature review, and review and editing. **Seyedeh Nasim Mirbahari:** writing original draft, supervision, data visualization, and investigation. All authors read and approved the final manuscript.

## Ethics Statement

The authors have nothing to report.

## Consent

For this type of study (Narrative Review), formal informed consent is not required as no new patients were recruited and no identifiable patient details or images were used beyond what is already available in the cited literature.

## Conflicts of Interest

The authors declare no conflicts of interest.

## Data Availability

All data generated or analyzed during this study are included in this published article.
